# A Systematic Review of Co-Educational Models in School Handball

**DOI:** 10.3390/ijerph182111438

**Published:** 2021-10-30

**Authors:** Ana R. Arias, Diego Soto, Camino Ferreira

**Affiliations:** 1 Research Group: (ÉVORI) Assessment, Guidance and Educational Policies, Department of General and Specific Didactics and Educational Theory, University of León, 24007 León, Spain; ana.arias@unileon.es (A.R.A.); camino.ferreira@unileon.es (C.F.); 2 Department Physical and Sport Education, University of León, 24007 León, Spain

**Keywords:** handball, sport, gender, physical education, inclusion

## Abstract

This study aims to address the characteristics presented by the co-educational models that have been put into practice in school handball, showing a general overview, after providing a systematic review of the literature on the topic published over the last ten years on co-educational paradigms in the practice of handball as a school sport. For the description and recording of the process of selection and filtering of documentary sources to be analyzed, use was made of the PRISMA flowchart. After the elimination of duplicates and entries not compliant with the criteria for time limits and type of document, the final sample surveyed was composed of thirty academic articles. The results considered (*n* = 26) showed a presentational pattern divisible into three segments or groups. These were: technical and sporting aspects of handball, highlighting the benefits of this sport in schools relative to other options (*n* = 7), co-education (*n* = 8), and results based on differences between the sexes (*n* = 11). Co-education stresses significant improvements in skills, together with perceived effort, enjoyment, and participation. These lead to improvements in the social climate and group cohesion, thanks to the practice of sports. Handball within schools, seen from a co-educational angle, should be approached with an eye to its predisposition for the development of social and civic skills. These include respect for the rules of a game, teamwork and solidarity, fair play, commitment, and responsibility. This paper lays out precisely and exhaustively the lines of investigation undertaken in the area of co-education, and, more specifically, how this is handled within the practicing of a confrontational team sport like handball.

## 1. Introduction

Many may think that it is a challenge that has been overcome, but reality shows that, in a number of areas of life, inequality between the sexes continues to be obvious, particularly in the educational context [1,2]. The geographical context influences this matter [3,4,5]. This is so much the case that in the field of Physical Education, where the physiological differences between the two sexes are clearly visible, educational professionals should take an active part in developing the qualities of individuals and boys dominate the space and the teachers only attend to the “sports girls” [6]. They should always remain aware of the fact that this means working with two different sexes that grow up, despite sex-based barriers, in a shared social reality marked by androcentric structures in Physical Education [7]. This would be the definition of the term co-education.

The strong intrinsic view, based on physiological characteristics, defining sex inequality, is accompanied by other, no less crucial variables, such as socio-cultural and relational factors, and personal beliefs. It is possible to go beyond a merely physical, bodily viewpoint and take into account the part played by the education system, and not just personal sexual characteristics, in terms of the provision of resources and equality, or the lack of it, in the system itself [2,8]. Education should address itself to the integrated individual development of pupils on the basis of Universal Design for Learning (UDL), which is to be seen as a theoretical and practical approach acting as an effective tool in striving for an education that will provide both quality and equality [9].

The aim would be to achieve an equitable teaching approach of this nature [10,11,12], incorporating into it a participatory angle [13]. A co-educational system aims at educating about values, through learning the positive and negative aspects of feminine and masculine models [14]. To this must be added an affective component [15], on the premise that emotions should receive an equalitarian treatment [16,17].

As it is a part of Physical Education, this article will go deeper into the field of school sports, and in particular the practice of handball in which physical education teachers indicate that it is an easier sport to teach [18]. This implies a format allowing generic application of what is explained here to any sports discipline [19], and naturally from a co-educational viewpoint. A range of shaping factors must be taken into account when holding sports sessions, these being evident in the “hidden curriculum” of any school [1,14]. In the light of this, it can be noted that teaching staff reveal their beliefs, which tend to be stereotyped [16] unconsciously and automatically [17,19,20,21]. However, these “hidden” views are not absent among pupils, either [7,22,23], since the social roles and values of the society in which they grow up are reproduced in them [24].

To return to the concept of a facilitator, it becomes necessary to analyze the teaching methods employed [2,25] or training in sports techniques [26,27]. In so doing, there should be a review of the conceptualizations taken on board about competitiveness, aggressiveness, or domination that are clear in models for sports, and which in general have connections with masculine standards [28]. This fact makes it all the more necessary to critically analyze the current P.E curriculum [29] and more research on coeducation [30].

Such a male-centered point of view has led to unequal use of spaces for movement and of material [31,32], for instance, by not keeping in mind the size and weight of balls as an excluding factor [33]. Likewise, little account has been taken of differing behaviors in the system of relationships that become established in play and school sports [34,35]. This has an impact on the sorts of groupings proposed by teachers, and also repercussions on the evaluation or feedback given, as a function of pupils’ sexes.

After this general panorama, the research question addressed was the characteristics presented by the co-educational models that have been put into practice in school handball. A systematic review of the literature has been carried out. This type of study makes it possible to review the literature on a topic, starting from a question and using systematic and explicit methods in the collection and analysis of data with the aim of highlighting the main conclusions on the topic. In this objective, the problem and the research question are clearly and specifically defined. For their definition, the PICO(C) strategy is used. Taking this strategy into account, the population (P) of women in the practice of handball is studied by means of the intervention (I) of a bibliographic search related to the objective in the selected databases, comparing (C) the practice of handball as a school sport, seeking as outcomes (O) to identify which coeducational models have been put into practice in handball in the school context (C). In this way, we will answer the research question: what are the co-educational models that have been put into practice in school handball?

The main aim was to present a general overview, after providing a systematic review of the literature on the topic published over the last ten years on co-educational paradigms in the practice of handball as a school sport.

The overview was to be achieved by fulfilling certain objectives, specifically:

- A description of the bibliography of studies published in relation to co-education and handball.
- The presentation of a mind-map of keywords representing the research work considered.
- An analysis of the purposes of the investigations was selected on the basis of a co-educational perspective of handball as a school sport.
- A summary of the principal results and conclusion of this analysis of publications relating to co-educational perceptions in school handball.

## 2. Methods

The method adopted for this study was a systematic review. Such a methodology is characterized by being valid, reliable, and repeatable. A systematic review of relevant literature makes it possible to judge the extent and depth of pre-existent work and to identify any gaps that require investigation [36].

The research followed the Preferred Reporting Items for Systematic reviews and Meta-Analyses (PRISMA) [37] guidelines determined as: (i) definition of the objectives with explicit and reproducible methodology; (ii) systematic search for evidence following eligibility criteria; (iii) assessment of the validity of the findings; and (iv) systematic presentation and synthesis of the characteristics and findings of the included studies.

(i) Definition of the objectives with explicit and reproducible methodology.

In the review carried out, consideration was given to academic articles concentrating on the topic under study: handball as a school sport, seen from a co-educational angle.

(ii) Systematic search for evidence following eligibility criteria.

In order to perform the systematic review, use was made of three major databases: Web of Science (including all its subsidiary databases: the main collections of Web of Science, Current Contents Connect, Derwent Innovations Index, the KCI Korean Journal Database, MEDLINE, the Russian Science Citation Index, and the SciELO Citation Index), Scopus and ERIC. In the light of the aims of the study, the search chain applied to these databases was the following: AB = (women OR girl * OR gender *) AND (sport OR physical activity OR physical education) AND school AND handball)

The inclusion criteria for this systematic review were:

- Type of document: Academic articles and reviews
- Time limit: 2010 to 2021 (up to April of this last year)
- Language of publication: English, Spanish, or Portuguese

The number of entries identified in the databases was 123. After the elimination of duplicates and entries not compliant with the criteria for time limits and type of document (for example, conference proceedings and books were ruled out), 109 entries were filtered. Of these, forty-eight were excluded and one proved impossible to retrieve. The sixty entries that emerged from the filtering stage were evaluated for eligibility, with thirty retained for the review and thirty excluded.

In the two phases of filtering, a total of seventy-eight articles were excluded because they did not concentrate on the topic under analysis. Specifically, there were exclusions on the grounds -first phase- of being related to high-level sport, Special Education, or university sport, to the training of teams, to the physiological dimension and injuries, or Conference-Congress-Minutes-Books. Other items -second phase- were discarded because they were connected to different objectives such as “tech-tach” (techniques and tactical skills), bullying, refereeing, and the like. Some entries were ruled out because they had no link with education or did not include females among their samples. Hence, the final sample surveyed was composed of thirty academic articles, shown in the bibliographic references section with a prefixed asterisk.

Entry selection was carried out using the Mendeley reference manager, while for the analysis of the studies included in the survey the qualitative analytic package MAXQDA Analytics Pro 2020 was employed. Bibliographic data were exported in Research Information Systems (RIS) standardized tag format through Mendeley and imported into MAXQDA, distinguishing the type of reference, title, main author, volume, issue, first and last page, publisher, digital object identifier (DOI), name of the journal, notes, website or uniform resource locator (URL) details, and date of publication.

Content analysis of the academic articles was on similar lines to those of [38]. Main analytic categories were established for the documents as a function of the objectives, results, and conclusions laid out in them.

(iii) Assessment of the validity of the findings

The methodological quality of the included studies was assessed using a risk-of-bias quality form of 7 items validated and adjusted for the specific context of co-educational research [39]: 1. Time period given. 2. Dropouts given. 3. Type of survey described. 4. Sex of participants given. Study design given and 7. Co-education mentioned. Each question was answered with a yes if the criteria were satisfied (2 points), with a don’t know (1 point), or with a no if the criteria were not satisfied (0 points). Based on this procedure, the studies were classified as follows: low methodological quality (≤50% of total points); good methodological quality (51–75% of total points); and excellent methodological quality (>75% of total points). Shows the scores for the quality assessment, values ranged from 12 to 14 points, with an average score of 12.67 points—90.5%. Regarding the individual quality assessment, all studies were categorized as excellent.

(iv) Systematic presentation and synthesis of the characteristics and findings of the included studies

**Figure 1:**
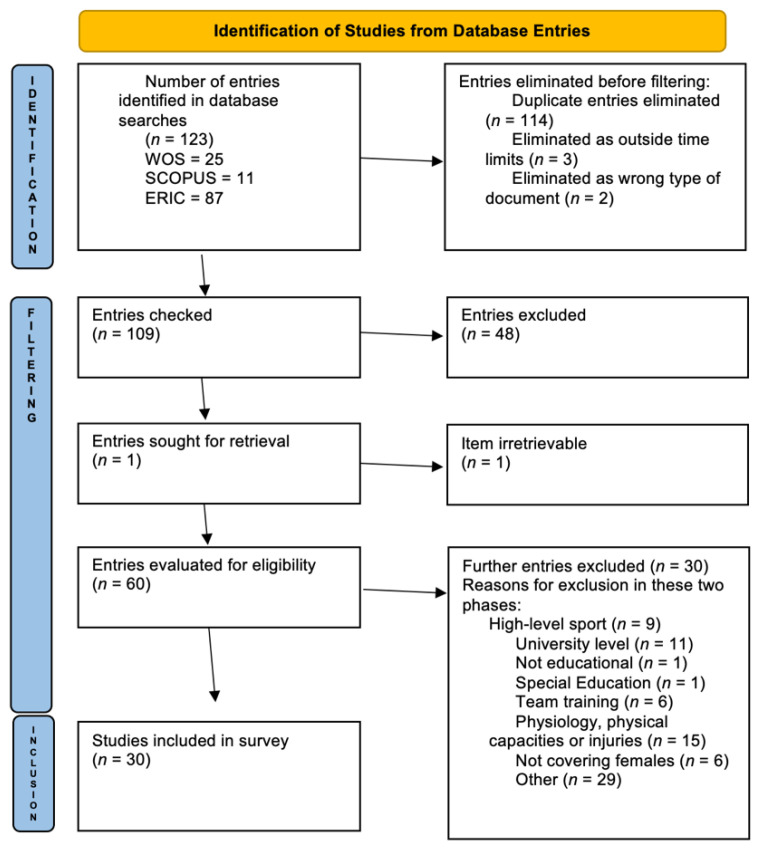
PRISMA Flowchart; Source: Drawn up by the author, following [37].

For the description and recording of the process of selection and filtering of documentary sources to be analyzed, use was made of the PRISMA flowchart. Such a diagram is split into three phases: identification, filtering, and inclusion, as shown in Figure 1.

## 3. Results

### Bibliographic Data and Principal Characteristics of Articles

In view of the inclusion criteria for the systematic review undertaken, all the entries analyzed were articles published in academic journals. Table 1 shows the main authors, the journals in which articles appeared, and the year of publication, which is the item used to order entries. With regard to principal authors, it was found that a couple of researchers had more than one item on this topic. These were the authors Farias (*n* = 3) and Puente Maxera (*n* = 2), whilst all the others had only a single article.

Consideration of the journals in which the academic articles covering the topic under study were published show that there was some concentration in a range of periodicals. Specifically, these were European Physical Education Review (*n* = 4), Physical Education and Sport Pedagogy (*n* = 4), Research Quarterly for Exercise and Sport (*n* = 2), Strategies: A Journal for Physical and Sport Educators (*n* = 2), The Physical Educator (*n* = 2) and Universal Journal of Educational Research (*n* = 2).

**Figure 2:**
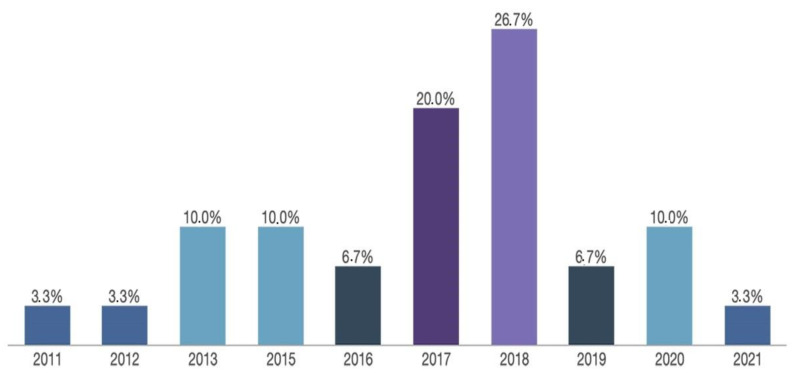
Progression among Publications Selected (2011 to 2021); Source: Compiled by author.

In respect of the year of publication, Figure 2 shows that there has been an increase in the number of articles published on this topic in recent years. This especially affects 2017 and 2018, when almost half of the publications appeared (*n* = 14).

**Figure 3:**
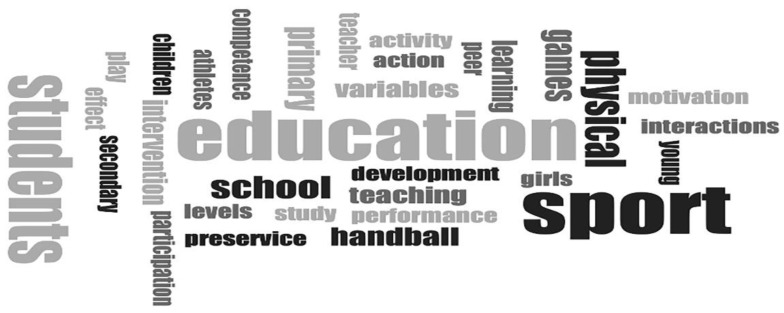
Word cloud of Words from Titles of Articles Selected; Source: Compiled by author.

The titles of the items published were also considered from the viewpoint of word frequencies, as shown in Figure 3. This figure provides a rapid general view of the terms most often in use. A word frequency analysis indicated that the term education is present in 57% of the article titles, whilst sport was to be found in 53%. Details of the intended audience were revealed by the use of students, school, and girls, and handball indicated the sport being analyzed in this study. The practice of this sport was tied in with teaching and learning, performance and development, action, interactions and activity, participation and intervention, games, and motivation.

These details are closely linked to those recovered from the contents of articles and the keywords chosen by their authors. This field of analysis shows a considerable range of varying segments (*n* = 138), a consequence of the generic nature of keywords, and in some cases an obligation to select words from among those included in lists imposed as a standard by the journals in which items were published.

This being accepted, the groupings run through Inclusive Education and the Sexes (*n* = 20), Teaching Techniques (*n* = 23), Research Methods (*n* = 8), Life Stages (*n* = 6), Educational Stages (*n* = 14), and Physical Education and Sport (*n* = 53) to Psychological, Physical and Behavioural Aspects (*n* = 22).

Those terms coded under the label Inclusive Education and the Sexes can be further broken down. Among other items, there are Roles and Social Status (*n* = 2), Development of Sex-Based Stereotypes (*n* = 1), Differences between the Sexes (*n* = 4), Literal Concepts of Inclusion (*n* = 1), and Co-education (*n* = 1), this last aspect being of note if it is recalled what topic of study is proposed here.

## 4. Analysis of Aims

**Figure 4:**
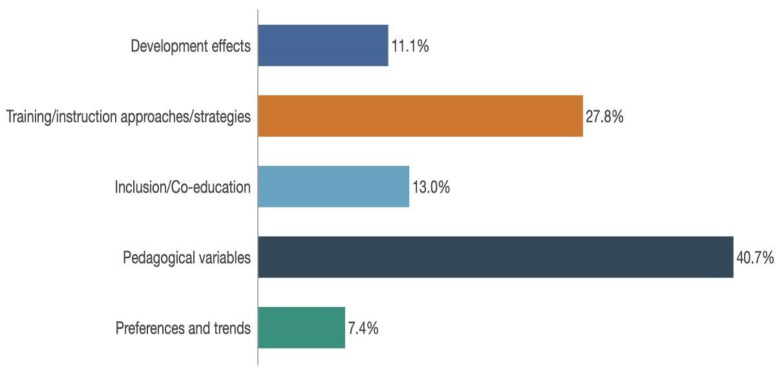
Weighting of the Main Objectives Envisaged; Source: Compiled by author.

Consideration of the objectives proposed for handball as a school sport from a co-educational perspective reveals that there are five major aims in the items surveyed (Figure 4). Items concentrating on pedagogical variables took pride of place (39.6%), followed by those evaluating approaches, training strategies, or instruction (28.3%). Only 13.2% of the articles had inclusion and co-education as their principal topic of study, whilst even smaller percentages of articles were to be found on the effects in developing education or physical activity (11.3%) and on preferences and trends in this area (7.5%).

Table 2 shows the coding system by aim and the quantity of articles picked. A major portion of the articles looked at mediation strategies for teachers, at the instruction of student trainers, and at developing pupils’ games (*n* = 15). Among these, some concentrated on fair play and sporting behaviour (*n* = 3), others on efficiency (n = 1), skill levels (*n* = 4), education for dynamic equilibrium (*n* = 1) and tactical game models or TGM (*n* = 1).

The second group of articles considered aspects related to inclusion and co-education (*n* = 7). These articles addressed democratic, inclusive, and participatory models for handball as a school sport, highlighting sex-related differences between males and females (*n* = 6).

Pedagogical variables were extensively analyzed, since handball was being studied in an educational context (*n* = 21). Among other matters, there was an exploration of participation by pupils in undertaking this school sport (*n* = 1), of the transition between stages of education (*n* = 1), of those involved, or in other words of the role of teachers or coaches (*n* = 3) and of the interaction between teachers and pupils (*n* = 1). There were investigations of the part played by spoken interchanges between team-mates when group sports are underway (*n* = 2), of the basic abilities and concepts to be taught in handball as a school sport (*n* = 5), and of how these may be reflected in lessons taught (*n* = 1). Within this range of pedagogical variables, certain aspects relating to motivation stood out (*n* = 6), whether from the angle of confrontation, or alternatively of cohesion and a motivational atmosphere.

Preferences for certain sports and trends among children towards given types of activity, as also sporting culture were also scrutinized. Nevertheless, this occurred to a lesser extent (*n* = 4) and was particularly related to handball as opposed to other alternative sports.

Finally, the developmental effects of practicing this school sport were also examined (*n* = 6). Specifically, these investigations referred to its impact on the immune system and psychology, to the development of motor skills, mind, and physical fitness, to cognitive and psychomotor development, and health, especially in relation to obesity and quality of life.

## 5. Analysis of Results 

The results considered (*n* = 26) showed a presentational pattern divisible into three segments or groups. These were: technical and sporting aspects of handball, highlighting the benefits of practicing this sport in schools relative to other options (*n* = 7), co-education (*n* = 8), and results based on differences between the sexes (*n* = 11).

Such differences between the sexes as were noted, allowance being made for social stereotypes, fell into three areas of analysis. First, there were the repercussions on actions, and technical and sporting roles (*n* = 3), whether these related to refereeing or coaching. A second field corresponded to differences of a physical and physiological nature between the sexes (*n* = 2). Finally, there were differences seen from the angle of psychological and behavioral aspects (*n* = 6).

Co-education (*n* = 8) stresses significant improvements in skills, together with perceived effort, enjoyment, and participation. These lead to improvements in the social climate and group cohesion, thanks to the practice of sports.

Carcamo [67] reports that boys/girls have a dominant gender narrative that makes femininity subordinate to masculinity, thereby encouraging binary gender beliefs and practices reaching the conclusion the need to make boys and girls more aware of gender equality, make changes to the activities and to how the physical spaces are used and provide equal teaching and learning experiences to reduce a divide still present in physical-sports education [61]. The PE curriculum alone was insufficient to dismantle the deeply rooted negative cultural influences of community-based sports that influenced equity and inclusion. However, by planning and implementing a specific intervention that used the educational resources of Sport Education proactively it was possible to promote a more inclusive and equitable learning environment.

In terms of participation, different studies indicate that girls are less active in traditional invasion sports because of their skills. Reference [70], translate into a sporting mindset [71]. Using mini-handball to assess the level of activity, it was found that to balance the activity, groupings should be made by skill level to achieve greater participation [51]. Another proposal combining sports such as handball with other so-called modern sports such as tchoukball increases participation [69]. On the other side, the students’ interpretation of their participation defines their future memories and, among other things, their adherence to sport in adulthood [72]. These strategies should be assessed in longitudinal studies because has implications for the high number of girls who drop out of the sport.

Moreover, with regard to the conclusions of the articles analyzed, it is possible to establish two major groupings. First, there are those codified segments that offer technical comparisons relative to other sports (*n* = 6 segments of 26 coded) with differences between the sexes being clear in the sports disciplines considered. Second, there is a large group comprising aspects directly linked to co-education and equality (*n* = 20), specifically views on the merits of educational methods (*n* = 11) and the physical and psychological benefits for females (*n* = 6), within an attempt to overcome learned social stereotypes. Among others, Kovács [48] concludes that during the match there is a significant difference between the perceived anxiety against hard and weak teams at the beginning, in the middle, and at the end of the match; furthermore, females have a higher level of anxiety during the match. Again, the scientific evidence shows the necessary inclusion of co-educational strategies. School stands out as a sustaining element for a new line of educational activities in which equality between the sexes can constitute an enriching tonic for society.

## 6. Conclusions

At this point, there should be some reflection on the extent to which the intended objectives of this research were attained. This paper lays out precisely and exhaustively the lines of investigation undertaken in the area of co-education, and, more specifically, how this is handled within the practicing of a confrontational team sport like handball.

Handball within schools, seen from a co-educational angle, should be approached with an eye to its predisposition for the development of social and civic skills. These include respect for the rules of a game, teamwork and solidarity, fair play, commitment, and responsibility.

This study has pointed up the main fields of analysis of co-education and handball as a school sport. These include a need to enquire into pedagogical variables, an evaluation of approaches, training strategies, educational equality and inclusion as a motor for change, and the beneficial effects of physical activity, whether on a bodily, a psychological, or a behavioral plane.

Handball is seen not merely as a sport, but a tool to be used in presenting educational strategies aimed at equality, since the deep-seated conception of taking part in sports is tied up with attitudes that are socially and culturally imposed, because of their competitive nature. Teachers, coaches, and pupils should be aware of a need for change.

As for the topic being addressed here, the most important conclusion that can be drawn relates to the positive repercussions of sports education upon co-education and equality in education. This aspect becomes all the more prominent when it is realized that handball is the third commonest physical activity among sports practiced at school, especially by girls, although it must be noted that it is not so often played outside a school context [38].

In respect of the use of materials and equipment in an educational context that would encourage participation by girls, no research was found that addressed this theme. In view of the pioneering work by Oliver, Sosa, and Porras [33] that pointed to a need to adjust and graduate possible differences in the proportions of balls to be used in handball by females and by males, also taking into account the age of participants, there would appear to be some requirement for further research that would determine the effects that the use of appropriate material would have on participation in sport by female pupils.

On the premise that there is a need for methodological change if heightened awareness on the part of children of equality between the sexes is to be achieved [60], certain strategies should be adopted. These include negotiation [46,68] and active problem resolution [40,49], which stand out as techniques favoring the inclusion of female pupils in the practice of sports. On the other hand, there is a great lack of knowledge in reference to the specific methodology for teaching handball that aims at a better integration and satisfaction of the girls when they play handball. In addition to this, it is necessary to know with greater accuracy the influence of the teacher’s gender, since the female gender uses games more frequently as a means of teaching handball, which implies a higher level of opposition and a greater number of subjects involved in the task [50].

Moreover, the benefits observed from taking part in school sport with regard to psychological, physical, and behavioral aspects have a positive impact on the perceived skills of girls [41]. They also enhance the extent of intrinsic motivation and reduce anxiety levels [48].

Several limitations were noted in this survey, and the chief among them are highlighted hereafter, alongside details of prospective research that would be a means of palliating them. First of all, there was a number of works found and information overload, leading to a need to summarize, which may have caused the loss of some relevant information that should be taken into account in future studies. Likewise, “grey” literature, material not published through conventional academic or commercial routes, has been omitted, even though it might be a suitable object for study in view of the applied nature of the topic.

As in all literature reviews, any selection of articles can be seen as introducing a bias that constitutes a limitation. Similarly, there is a constant necessity to update such surveys as time passes.

The line taken in this work was intended to diagnose the current state of affairs in co-education. This included investigation of the syllabuses for the compulsory stages of education that have an impact on the Physical Education curriculum and sport outside school. It also looked at real practices in handball, analyzing the methodologies for teaching and learning that are applied and the concepts of facilitators and pupils. As a function of the results, this should permit the development of specific action plans aimed at improving the quality of education from the twofold perspectives of the sexes and of sport. An outcome would be the application of teaching strategies relating to the use of material, modulations in physical contact, groupings, and playing areas. For this, it would be necessary to create methodological guidelines to help teacher-coaches to achieve an attractive sporting experience that includes the specific needs of girls.

The curricular trend recorded relates directly to an analysis of co-educational policies. Participation in sports during the school years is linked to the world of work as adults, in which there are parallel roles to those of trainers, managers, and others. Of particular note that one of the major limitations in reaching conclusions was the lack of longitudinal articles showing the evolution of the different aspects analyzed. The effects of the implementation of co-education should be observed over time, among others, by reducing sports drop-out rates and occupying leadership positions in sports that are mostly occupied by the male gender [73].

This paper cannot end without accepting that there is still a long way to go. It has shown that there was an upswing in the amount of literature in existence noted in the survey in relation to the years 2017 and 2018, even though the focus was exclusively on handball. This points to the opening up of a line of action attempting to anchor itself in schools as a basis for social progress. Schools and school sport should inculcate social values, equality, fairness, respect for differences, and in particular an understanding of individuals that goes beyond seeing no more than one distinguishing feature, their sex. In brief, they should reduce inequalities and aim to achieve tolerant societies.

## Figures and Tables

**Table 1:** Description of Articles Analysed.

Main Author	Journal	Publication Year	Coding Segments
Lima da Silva et al. [40]	*Movimento*	2011	22
Abernethy et al. [41]	*Journal of Experimental Psychology: Applied*	2012	30
Slingerland et al. [42]	*European Physical Education Review*	2013	31
Eys et al. [43]	*Research Quarterly for Exercise and Sport*	2013	30
Wallhead et al. [44]	*Journal of Teaching in Physical Education*	2013	31
Farias et al. [45]	*Sport, Education and Society*	2015	25
Darnis and Lafont [46]	*Physical Education and Sport Pedagogy*	2015	25
Phillips et al. [47]	*Strategies: A Journal for Physical and Sport Educators*	2015	24
Kovács, Nagy et al. [48]	*Practice and Theory in Systems of Education*	2016	33
Ramos and Esslinger [49]	*Strategies: A Journal for Physical and Sport Educators*	2016	22
Gamero et al. [50]	*e-balonmano.com: Revista de Ciencias del Deporte*	2017	29
Hastie et al. [51]	*Physical Education and Sport Pedagogy*	2017	22
Brock and Hastie [52]	*European Physical Education Review*	2017	31
Demirci et al. [53]	*Universal Journal of Educational Research*	2017	28
Kristiansen and Stensrud [54]	*British Journal of Sports Medicine*	2017	30
Lopes et al. [55]	*Physical Education and Sport Pedagogy*	2017	31
Farias et al. [56]	*Research Quarterly for Exercise and Sport*	2018	28
Güler [57]	*European Journal of Physical Education and Sport Science*	2018	24
Puente-Maxera et al. [58]	*Cultura, Ciencia y Deporte*	2018	20
Schwamberger and Curtner-Smit [59]	*The Physical Educator*	2018	25
Adé et al. [60]	*Physical Education and Sport Pedagogy*	2018	30
Farias et al. [61]	*European Physical Education Review*	2018	29
Hodges et al. [62]	*The Physical Educator*	2018	36
Üzüm [63]	*Journal of Education and Training Studies*	2018	29
Puente-Maxera et al. [64]	*Ágora para la Educación Física y el Deporte*	2019	26
Kucukibis and Gul [65]	*Universal Journal of Educational Research*	2019	28
Adigüzel [66]	*African Educational Research Journal*	2020	23
Cárcamo et al. [67]	*Human Arenas*	2020	23
McEntyre et al. [68]	*European Physical Education Review*	2020	29
Robles Rodríguez and Robles Rodríguez [69]	*Retos*	2021	23

Source: Compiled by author.

**Table 2:** Objectives of the Selected Articles.

Coding System	*n*	Articles
Approaches/Strategies for Training/Instruction	5	Abernethy et al. (2012) [41]; Farias et al. (2018) [56]; Farias et al. (2018) [61]; Ramos and Esslinger (2016) [49]; Wallhead et al. (2013) [44]
Fair Play and Sporting Behaviour	3	Ramos and Esslinger (2016) [49]; Schwamberger and Curtner-Smit (2018) [59]; Üzüm (2018) [63]
Efficiency	1	Lopes et al. (2017) [55]
Skill Levels	4	Adé et al. (2018) [60]; Hastie et al. (2017) [51]; Lima da Silva et al. (2011) [40]; Slingerland et al. (2013) [42]
Education for Dynamic Equilibrium	1	Adigüzel (2020) [66]
Tactical Game Models	1	Hodges et al. (2018) [61]
Inclusion/Co-education	1	Farias et al. (2015) [45]
Beliefs/Sex-Based Differences	6	Cárcamo et al. (2020) [67]; Kovács and Nagy et al. (2016) [48]; Kucukibis and Gul (2019) [65]; Puente-Maxera et al. (2019) [64]; Robles Rodríguez and Robles Rodríguez (2021) [69]; Slingerland et al. (2013) [42]
Paedagogical Variables	2	Adé et al. (2018) [60]; Gamero et al. (2017) [50]
Participation	1	Wallhead et al. (2013) [44]
Lessons Taught	1	Gamero et al. (2017) [50]
Role of Teacher/Coach	3	Farias et al. (2018) [45]; Schwamberger and Curtner-Smit (2018) [59]; Üzüm (2018) [63]
Basic Abilities and Concepts	5	Farias et al. (2018) [61]; Phillips et al. (2015) [47]; Puente-Maxera et al. (2019) [64]; Ramos and Esslinger (2016) [49]; Üzüm (2018) [63]
Teacher-Pupil Interaction	1	McEntyre et al. (2020) [68]
Confrontation and Motivation	4	Adé et al. (2018) [60]; Kovács and Nagy et al. (2016) [48]; Kucukibis and Gul (2019) [65]; Wallhead et al. (2013) [44]
Spoken Interchanges	2	Brock and Hastie (2017) [52]; Darnis and Lafont (2015) [46]
Cohesion and Motivational Atmosphere	1	Eys et al. (2013) [42]
Transition	1	Kristiansen and Stensrud (2017) [54]
Preferences and Trends	3	Güler (2018) [57]; Puente-Maxera et al. (2019) [64]; Robles Rodríguez and Robles Rodríguez (2021) [69]
Type of Sport (Individual or Team)	1	Kucukibis and Gul (2019) [65]
Developmental Effects	1	Farias et al. (2018) [45]
Immune System and Psychology	1	Kovács and Nagy et al. (2016) [48]
Development of Motor Skills and Physical Fitness	1	Lopes et al. (2017) [55]
Cognitive and Psychomotor Development	1	Phillips et al. (2015) [47]
Health	2	Demirci et al. (2017) [53]; Puente-Maxera et al. (2018) [58]

Source: Compiled by author.
